# Medical Principles

**Published:** 1858-10

**Authors:** 


### MEDICAL PRINCIPLES.

THERE are certain general principles in medicine, few and simple, easily understood, and remembered without difficulty; but instead of mastering these, the masses prefer to lumber up their memories with innumerable, and often ridiculous applications of them. We name one at this time.

POULTICES are of very extended application; that of a live chicken cut open and applied instantly, is believed by some to possess extraordinary virtue; the entrails of a frog are in great esteem by others; but how a frog is to be obtained in winter, is not explained; while a live chicken would be rather an expensive application in a city. Scraped potatoes are advised by some as possessing very great "drawing" powers. Dogs get well of their wounds without poultices. Dogs were the doctors who attended LAZARUS; and what is more than can be said of some modern doctors, they treated him as they did themselves, and, no doubt, with advantage. Dogs lick their sores. It is the instinct of Nature. Now let us not run off in the manner of the ignorant and superstitious, and imagine that there is some mysterious virtue in a dog's tongue, some magnetic agency passing back and forth; but let us look with our eyes open, and in the light of a few well-known facts. Take a common boil, about which most of us have had a feeling experience—the surrounding skin is dry, hot, and hard; in its natural state, it is moist and soft. To bring it to its natural state again, we have only to remove the dryness, and reduce it to its natural temperature, and what more appropriate than simple soft water, rained or distilled. But to get the full virtue of the application, it should be kept moist all the time; this would require constant attention, the incessant applying of water, which is in a measure impracticable, except to the unfortunate few who have nothing to do. The humane surgeon is often called to the exercise of a considerateness, which a large heart soon learns in its frequent contact with poverty, misfortune, and disease—it is in adapting his prescriptions to the means of the invalid, as far as possible; he never advises the worn and wan sewing-girl to visit Saratoga, nor the consumptive day-laborer to go to the South, or spend a winter in Cuba! so in the case of one suffering great pain, which can be best cured by keeping it constantly moist, he does not order a nurse who shall stand by and apply water from morning until night, but he bethinks him, that some substances do not dry up as soon as water (and which is, out of large towns, almost as easily had), one of these he knows to be milk; but even milk will require too steady an attendance, so he adds stale bread to it, which would retain the moisture still a longer time; and experience has found, that under all ordinary circumstances, a poultice of sweet milk, and stale "light bread," answers a larger number of conditions than any other, with the advantage of being always at hand in ordinary households. The softest fluid in nature is the saliva; and it is in this property we find the virtue of the dog's licking. But should the moistening substance be applied cold or warm? we must inquire of science. All know that the virtue of a cold or shower bath is in the reaction—the "glow," as it is called, which is nothing more than the rushing of blood to the surface in unnatural quantity and force; but the trouble in a boil or other sore is, that there is too much blood there already; instead of bringing more there, it is important to relieve it of its present load. So if a cold poultice is applied to a painful sore, besides the unpleasantness of the shock, there is the reaction inevitable as a consequence of that shock; therefore, Nature seems to teach us that all applications to painful sores should be moist, and soft, and warm, and constant; the dog's tongue answers all these requirements, and so does a warm milk-and-bread poultice.

But there is another beautiful physiological truth in warm applications, wherever there is inflammatory action, which it will be instructive to look at. It is evaporation which cools. But heat must precede evaporation. Ice-cold water does not evaporate, until wamth is applied; this requires time: hence, if warm water is applied to an inflamed surface, evaporation commences instantly, and proceeds rapidly. But in the case of a boil, what is evaporated, what is carried away? Two things. First, the heat. If the hand is put in warm water, and then exposed to the atmosphere, a sense of coldness is present; it is because the hand is parting with its warmth, giving it up to the vapor of water which is rising from the hand and passing upwards. But another effect is produced. A dry, hot skin, retains all beneath it, because the pores are closed; but as soon as it is moistened, the pores open, and at once the more gaseous and watery portions of the blood escape—thus reducing the bulk of the blood, reducing the distension, and thus reducing the pain.

In Eastern countries, and in earlier ages, where water in many places, and under many circumstances, was not very accessible, another substance was used; and its employment became almost universal for wounds, and bruises, and putrifying sores—and that was oil. For aught we know, the whole Materia Medica of ancient Surgery was wine and oil—wine, to sustain; and oil, to soften, and moisten, and lubricate, and cool. The great practical truth in all this is: the first necessity of a boil, or sore, or wound, is to keep it moist; that keeps down inflammation, pain, mortification, and death. To do this, a plain milk-and-bread poultice is the best, being accessible, simple, and safe—to say nothing of the advantage it has over many others, that it may be so readily re-moistened, and thus cleaned off.

